# Is the mouse nose a miniature version of a rat nose? A computational comparative study

**DOI:** 10.1016/j.cmpb.2024.108282

**Published:** 2024-06-08

**Authors:** Zhenxing Wu, Jianbo Jiang, Fritz W. Lischka, Kai Zhao

**Affiliations:** a Department of Otolaryngology-Head & Neck Surgery, the Ohio State University, 915 Olentangy River Road, Columbus, OH 43212, United States of America; b Monell Chemical Senses Center, Philadelphia, PA, United States of America

**Keywords:** Mouse olfactory, Nasal cavity, Micro-CT, Computational fluid dynamics, Odorant transport, Gas chromatography

## Abstract

**Background and objective:**

Although the mouse is a widely used animal model in biomedical research, there are few published studies on its nasal aerodynamics, potentially due to its small size. It is not appropriate to assume that mice and rats’ nasal structure and airflow characteristics are the same because the ratio of nasal surface area to nasal volume and body weight is much higher in a mouse than in a rat. The aim of this work is to use anatomically accurate image-based computational fluid dynamic modeling to quantitatively reveal the characteristics of mouse nasal airflow and mass transport that haven’t been detailed before and find key differences to that of rat nose, which will deepen our understanding of the mouse’s physiological functions.

**Methods:**

We created an anatomically accurate 3D computational nasal model of a B6 mouse using postmortem high-resolution micro-CT scans and simulated the airflow distribution and odor transport patterns under restful breathing conditions. The deposition pattern of airborne particles was also simulated and validated against experimental data. In addition, we calculated the gas chromatograph efficiency of odor transport in the mouse employing the theoretical plate concept and compared it with previous studies involving cat and rat models.

**Results:**

Similar to the published rat model, respiratory and olfactory flow regimes are clearly separated in the mouse nasal cavity. A high-speed dorsal medial (DM) stream was observed, which enhances the delivery speed and efficiency of odor to the ethmoid (olfactory) recess (ER). The DM stream split into axial and secondary paths in the ER. However, the secondary flow in the mouse is less extensive than in the rat. The gas chromatograph efficiency calculations suggest that the rat may possess a moderately higher odorant transport efficiency than that of the mouse due to its more complex ethmoid recess structure and extensive secondary flow. However, the mouse’s nasal structure seems to adapt better to varying airflow velocity.

**Conclusions:**

Due to the inherent structural disparities, the rat and mouse models exhibit moderate differences in airflow and mass transport patterns, potentially impacting their olfaction and other behavioral habits.

## 1. Introduction

As a main airway entry, the nose serves critical physiological functions: (1) the nose acts as the frontline to filter out harmful airborne chemicals and particles, (2) it warms and humidifies the inspired air, serves to protect the delicate lower airway and preserves body water, (3) airborne chemicals bind to receptors in the olfactory mucosa or trigeminal nerve endings in the nose to initiate chemical senses. The structure of the nose is closely related to its function. In terrestrial mammals, the nose’s internal structure includes a complex system of bone scrolls and plates called turbinates. Covered with mucus and epithelium, the turbinates act as a large surface area for capturing airborne particles and chemicals. They also facilitate heat and gas exchange and house millions of olfactory receptors. However, the turbinates also cause the nasal airflow to divide into various channels, making it more complicated to investigate. Understanding the uptake and transport of heat, water vapor, particles, and chemical molecules in the nose and their relationship to the anatomical structures is essential to comprehend the physiological functions of the nose fully.

Details of nasal airflow profiles have been investigated in humans [1–5], monkeys [6–8], rats [9–12], rabbits [13], canines [14–16], and felids [17,18]. Understanding nasal airflow has been crucial in comparing nasal airway anatomy and physiology interspecies. For example, the ethmoturbinates and the coverage of olfactory epithelium of macrosmatic species (rodents and dogs) are far much more convoluted than microsmats (humans) [19], which, as a result, is better for trapping airborne odorants [20]. The more efficient nasal absorption would lead soluble toxic gases (e.g., Formaldehyde) to have much higher incidents of nasal carcinoma in rodents than in humans, potentially causing a greater risk to lower airways in humans [11]. Data from laboratory animals, including mice, is crucial for extrapolating information about humans, as conducting some of the studies on humans is impossible. Dosimetry and pharmacokinetic models based on nasal airflow calculations in rats, rabbits, and monkeys have been developed to extrapolate human exposure guidelines [6,7,9,10,13].

**Fig. 1.**
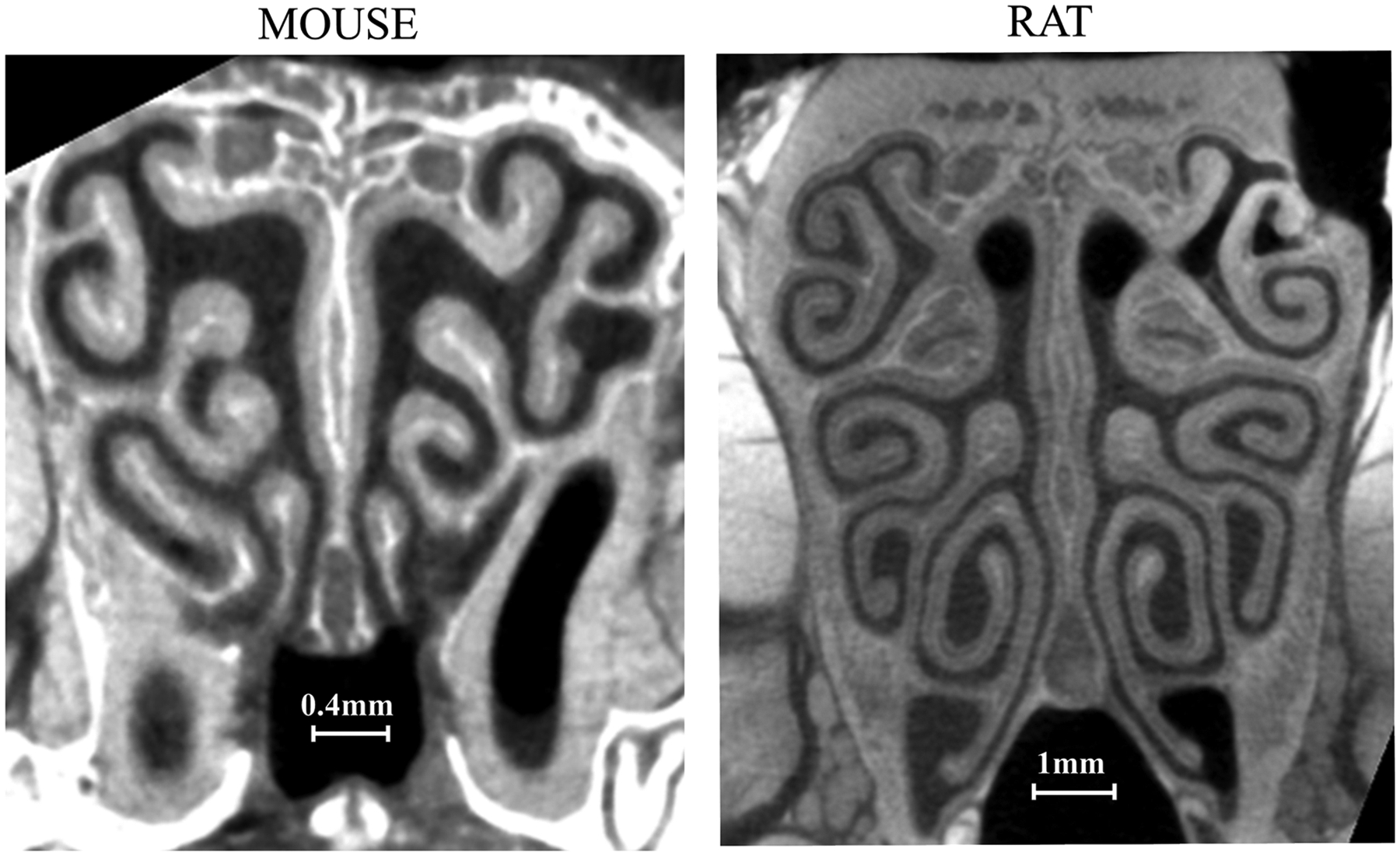
Micro-CT scans showing the mouse and rat’s nasal cavity cross sections. The dark areas visible on the scans represent the nasal airways.

The mouse, one of the most crucial animal models in biomedical research, has been used extensively in the study of molecular and neurophysiological mechanisms of olfaction [21–23], behavior [24], inhalation toxicology [25,26], including nasal lesions [27] and particle deposition [28,29]. Nasal airflow in mice cannot simply be assumed to be similar to that in rats since there are also marked differences in anatomy and physiology (Fig. 1). Gross et al. [30] showed that the nasal epithelium area of 7 and 16 weeks male mice (B6C3F1) is more extensive than rats (F-344) relative to body weight at the same age, whereas the latter has a more considerable absolute value of nasal epithelium area and volume. Under restful breathing, the respiratory frequency in mice (about 3~5 Hz) is higher than that in rats (~2 Hz), while the nasal airflow rate in mice (about 25 mL/min) is much lower than that in rats (~200 mL/min) [24,31–34]. The differences in nasal airway anatomy and respiratory parameters between rats and mice may translate to different airflow dynamics and possible functional differences.

Unfortunately, little information on the mouse nasal airflows has been reported, except for global airflow parameters such as nasal resistance [35] and point pressure signals [24]. Coppola et al. and Tatsunori et al. [36–39] have independently developed computational fluid dynamic (CFD) models for the mouse nasal airway. Still, their research is mainly limited to only one aspect of aerodynamics (odor absorption [36] or pressure drop [39]). The lack of detailed nasal airflow analysis for mice may be due to the much smaller, while possibly equally complex, anatomical nasal structures of a mouse than a rat, which makes the fabrication of scaled nasal replicas or computational fluid dynamics models based on the magnetic resonance imaging (MRI) or computed tomography (CT) scans difficult. The thinnest airway size in the olfactory region is approximately 40 μm in a mouse, 70 μm in a rat [40], and 200 μm in a cat [17]. MRI can increase spatial resolution by using specialized probes for small animals and prolonged scanning to enhance signal-to-noise ratio. The best spatial resolution found in references was 25 μm [36,37] under long scanning hours. Micro-CT can produce much better resolution in less time; however, CT cannot differentiate soft tissue with water or any mucus or liquid that may accumulate in the airway either in vivo or in vitro. Dye is often needed to increase the contrast [17].

This paper aims to utilize contrast-enhanced high-resolution micro-computed tomography (micro-CT) to develop a mouse nasal CFD model with a more detailed analysis than previously published. A numerical study of the mouse nasal airflow under physiological flow rate will be conducted to acquire a fundamental understanding of the mouse nasal aerodynamics relevant to its nasal function, compared to that of a published rat model. Based on the airflow simulation, We’ll further compare the odor transport efficiency of the mouse olfactory region with that of the cat [17] and rat models to further test the hypothesis that the ethmoid turbinates function as a parallel gas chromatograph to improve odor processing. It is evident that mass transport depends on nasal airflow patterns and features, which can provide additional functional relevance to the observed airflow patterns and features. Furthermore, particle transport deposition study will be performed and compared to experimental data in the literature, which, in addition to its functional relevance, serves importantly to validate the model.

## 2. Material and methods

### 2.1. The mouse specimen

An animal euthanized for reasons unrelated to this study was obtained. It was a B6 mouse, male, approximately 21 g in weight, six weeks of age, previously obtained from Jackson lab (B6129PF2/J). The head was removed and cleaned, then fixed in 4 % paraformaldehyde overnight.

### 2.2. Micro-CT images

It is well known that micro-CT provides excellent details of bone structure and air-tissue interface yet has low contrast between tissue and buffering water solution. However, the specimen cannot be thoroughly dried, especially during a scan that may last a few hours, which would otherwise result in significant tissue shrinkage and damage [17]. A Lugol solution has been used as a contrast-enhancing dye. The osmolality of the lugol solution was adjusted to match that of the plasma to reduce tissue shrinkage. A subsequent histology study confirms that the dye does not damage the tissue or interfere with subsequent histology dyes [17].

The fixed mouse head was submerged in 25 % diluted Lugol solution and rinsed out with PBS (phos buffered saline) immediately (minutes) before scanning. The specimen was then wrapped and sealed with parafilm (Parafilm Nursery Grafting Tape-Oesco, Inc.) before being placed in a Viva CT 40 (Scanco USA, Inc.) micro-CT scanner [17]. The scan took 4 h and obtained 3300 isotropic images with 2048×2048 pixels each (10.5 μm/pixel), using a bulb setting of 70 kV and 114 μA. A median filter with a 5 × 5 × 7 mask was applied to remove image noise while preserving the airway-mucosa edge (Fig. 1).

### 2.3. Nasal model and mesh generation

**Fig. 2.**
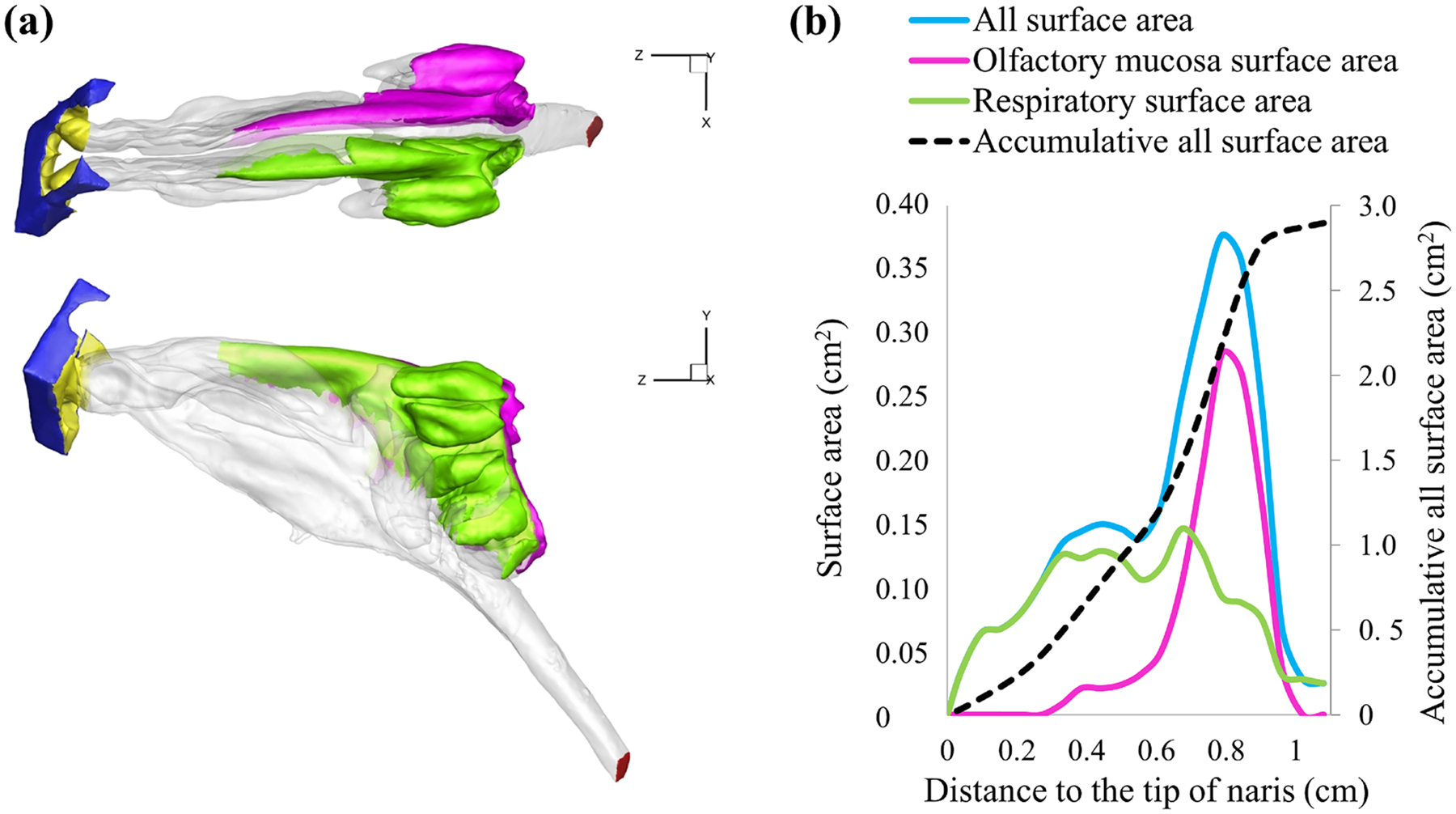
(a) 3D nasal airway model shows the distribution of respiratory (clear) vs. olfactory epitheliums (purple and green). (b) The surface area of the respiratory (green line), olfactory epithelium (purple line), and overall surface (blue line) as a function of distance from the tip of the naris. The black dashed line represents the accumulative surface area starting from the tip of the naris and integrated along the distance.

The mouse’s nasal cavity, similar to that of a rat, consists of the portion of the upper respiratory tract from the external nares to the nasopharynx from anterior to posterior. The nose is structurally separated into two equal compartments divided by a delicate cartilaginous nasal septum, including the nares, the turbinates, and the nasopharynx [41]. The compartments of the nose merge at a narrow opening called the septal window, located at the base of the nasal septum and anterior to the nasopharyngeal meatus [42]. Within the nasal cavity are three sets of turbinates from anterior to posterior: naso-turbinate, maxillo-turbinate, and ethmoid turbinate. The caudal ethmoid turbinates are much more complex in structure than the other two turbinates and are highly scrolled [41]. The olfactory region of the mouse nasal cavity is located dorsoposteriorly and is composed of ethmoid turbinates and part of nasoturbinates (Fig. 2a).

**Fig. 3.**
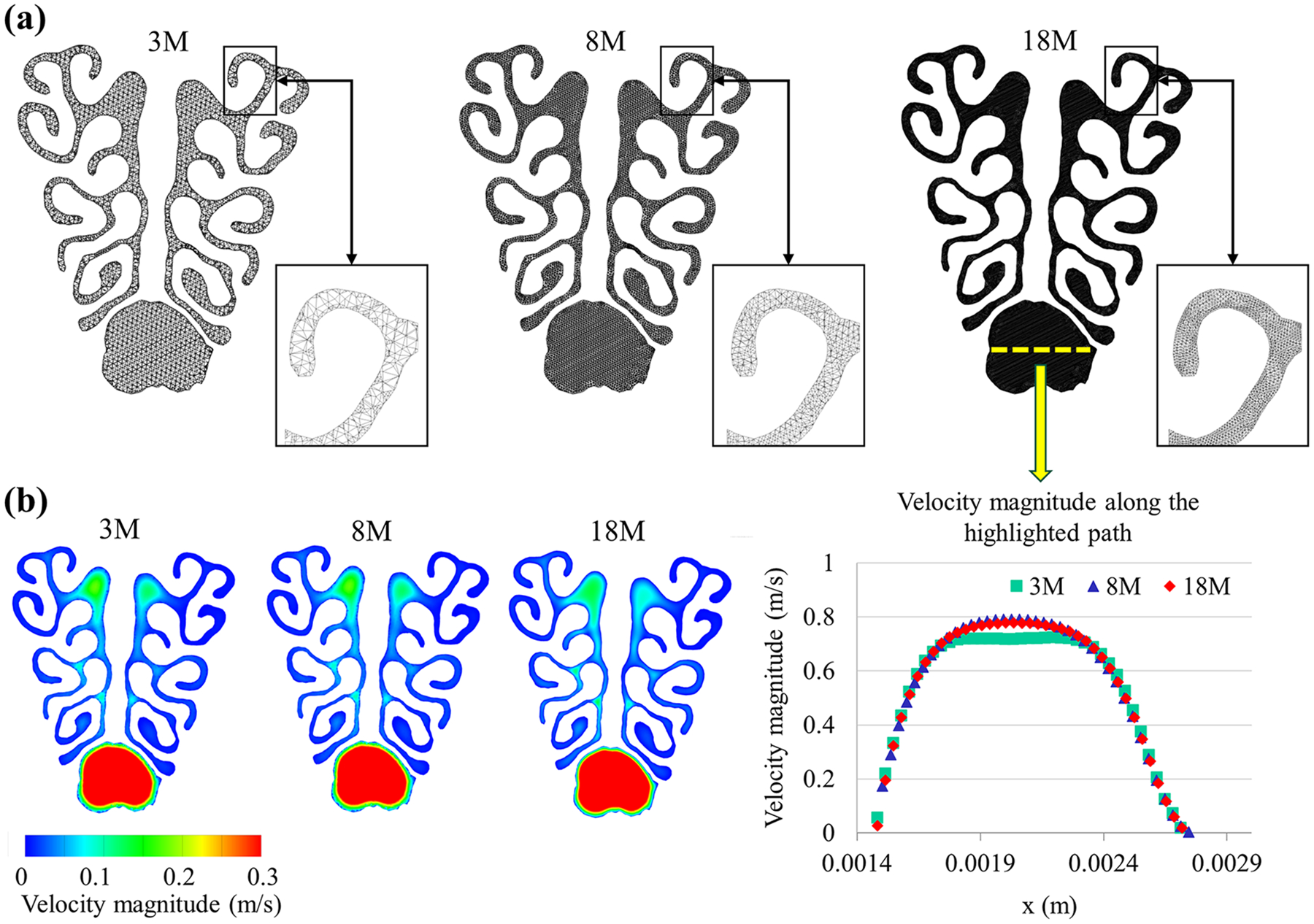
Grid independence (a) was verified by increasing the number of meshes. (b) It was observed that there was no significant change in velocity when the mesh was refined from 8 million to 18 million. Therefore, the results from the models that used 8 million grids were considered throughout the study and will be presented accordingly.

The AMIRA software (Visualization Sciences Group, Hillsboro, OR) was utilized to create a 3D model of the mouse nasal cavity based on the micro-CT images of the pretreated mouse specimen. This was accomplished through careful inspection, necessary smoothing, and artifact correction [17,20,43]. The resulting 3D model was an anatomically accurate representation that included the nasal cavity and nasopharynx, as depicted in Fig. 2a. The graph in Fig. 2b plots the surface area of various types of nasal epithelia as a function of distance from the nostril based on the created 3D model. A second software package (grid generator), ICEM (ICEMCFD, Engineering Inc., Berkeley, CA), was used to fill the 3D mouse nasal cavity model with tetrahedral elements [17, 20,43]. Grid independence was checked to ensure the accuracy of the study’s results. For grid refinement from 3 × 10^6^ to 1.8 × 10^7^ computational cells (Fig. 3a), it was observed that the alteration in the velocity fields was negligible from 8 × 10^6^ to 1.8 × 10^7^ (Fig. 3b). Thus, results from the models with 8 × 10^6^ grids (to balance model accuracy and computational expense) will be presented for all the studied cases except as specifically pointed out.

### 2.4. The rat model

A computational model of a rat nasal cavity previously published by our group using the CT scans of a Sprague-Dawley rat nose with similar geometry and mesh generation methods [40,44] was used in this study.

### 2.5. CFD simulations

A commercial CFD software, ANSYS Fluent 19.2 (ANSYS, Inc., Canonsburg, PA), was used to simulate the airflow pattern and odor transport inside the nasal cavity. The simulation method has previously been validated against experimental data [45,46].

#### 2.5.1. Airflow pattern

To simulate the nasal airflow, certain assumptions have been made. Firstly, it is assumed that the airflow in the mouse nasal cavity is incompressible, laminar, Newtonian, and quasi-steady [46]. Secondly, the nasal wall is assumed to be rigid, smooth, and with zero velocity (no-slip condition). These assumptions are commonly used in studies of nasal airflow [2,17,43,47,48], and Li et al. [46] validated that the laminar model achieved good agreement with experimental results using these assumptions under restful breathing conditions.

A uniform velocity was set at the inlet (nostrils), and a pressure outlet was used at the nasopharynx to achieve a restful breathing airflow rate of 25 mL/min and 200 mL/min for the mouse and rat models, respectively. The selected flow rate is based on experimental results [24, 31–34]. Specifically, the California Environmental Protection Agency’s Office of Environmental Health Hazard Assessment (OEHHA) has data on rat breathing rates [34]. The reasonable range for rat respiratory flow rate is 100–450 mL/min, and for mice, it is 10–35 mL/min. We selected median values for our CFD simulation.

The Womersley number (*W*_0_) [49] and the Strouhal number (*S*) [50, 51] were used to estimate the effects of unsteadiness on developing flows. The Reynolds number (*Re*) was used to estimate the impact of turbulence. In general, the assumption of quasi-steady flow is valid when the value of *W*_0_ is less than four, and the value of *S* is less than one [52,53]. On the other hand, the assumption of laminar flow is valid when the Reynolds number (*Re*) is less than 2300. In our rat model, the quasi-steady flow assumption can be applied to most rat breathing conditions [52]. For the mouse nasal model, the *W*_0_, *S*, and *Re* numbers are considerably smaller than those in the rat model (*Re*=15, *S*=0.46, *W*_0_=0.42, calculated on a cross-section near naris) due to the geometry size of the mouse nasal airway. Thus, the effects of unsteady and turbulent breathing are neglected in our current study. More details can be found in our previous research [17] and supplemental material S1.

#### 2.5.2. Odor transport

Similar to our previous research [17,44,45,52], the odorant concentration at the boundary of the air-mucus interface satisfied [47]:

(1) $$\frac{\partial C^{\prime }}{\partial y^{\prime }}+KC^{\prime }=0\;\text{with}\;K=\frac{d_{in}D_{m}}{D_{a}\beta d}$$

where *C*′ represents the odorant concentration, *y*′ represents the local coordinates perpendicular to the air-mucus interface, *d*_*in*_ is the hydraulic diameter (the diameter of nostrils); *D*_*m*_ and *D*_*a*_ are the diffusion coefficients of the odorants within the mucosa and air, respectively, which were calculated using Wilke-Chang equation [54]; β represents the air-mucus odorant partition coefficient [17,44,52]; *d* represents the mucosal layer thickness. The odorant mass transport, using the boundary condition of the air-mucus interface with adjusted *K* parameter (Eq. (1)), showed good agreement with experimental results in our previous study [45].

Various odorants (as listed in supplemental material S2 Table) were chosen to simulate odor transport and depositions within the mouse nasal cavity that may be relevant to its sense of smell.

### 2.6. Secondary flow

Secondary flows refer to the velocity components not aligned with the primary flow direction induced by the geometry of flow configuration and/or turbulence. They are essential for transporting or diverting mass (e.g., odorant molecules, particles, heat, vapor, etc.) away from the central stream to peripheral regions. The strength of the secondary flows (*SS*) is defined as the ratio of secondary flow velocity to the total flow velocity [52]:

(2) $$SS=\sqrt{\frac{v^{2}+w^{2}}{u^{2}+v^{2}+w^{2}}}$$

where *u* is the velocity component in the axial (z-axis, from nostrils to nasopharynx) flow direction, *v* and *w* are the velocity components in the plane perpendicular to *u*, which are in x-axis and y-axis directions, respectively.

### 2.7. Gas chromatography efficiency analysis

Our previous research [17] suggested that the mammalian olfactory turbinates could act as a coiled parallel gas chromatograph (GC) system to enhance its retention and processing of odor information. Here, we applied the same concept and analyzed the theoretical plate number (*N*) to evaluate the GC efficiency for the mouse and compare it to that of the rat and the domestic cat:

(3) $$N=L_{c}/H$$

where *L*_*c*_ is the column length, the average distance that an odor compound travels from the olfactory inlet to the olfactory outlet; *H* represents the plate height that can be determined by the Golay equation [55, 56]. The details of the calculation of the plate height can be found in our previous research [17] and supplemental material S1.

### 2.8. Particle deposition

Inhaled particle trajectories through the mouse nasal cavity were calculated using the Discrete Phase Model in FLUENT (ANSYS, Inc., Canonsburg, PA). The motion path of a discrete particle can be determined by integrating the force balance on the particle using a Lagrangian reference frame. This force balance can be written as [57]:

(4) $$\frac{du_{p}}{dt}=\frac{18\mu }{\rho_{p}d_{p}^{2}}\frac{C_{D}Re_{p}}{24}(u_{f}-u_{p})+\frac{g(\rho_{p}-\rho_{f})}{\rho_{p}}+F_{s}$$

where ***u***_***p***_ is the particle velocity, ***u***_***f***_ is the fluid phase velocity, ρ_*p*_ is the particle density, ρ_*f*_ is the fluid density, *μ* is the molecular viscosity of the fluid, *d*_*p*_ is the particle diameter, *C*_*D*_ is the drag coefficient, *Re*_*p*_is the particle Reynolds number [57], ***g*** is the gravity force, ***F***_***s***_ represents the Saffman lift force [12,57,58].

Eight thousand particles were released from the mouse nostrils for each particle size. To minimize the diffusion effect, the particle size considered in the current study is between 1.5 and 43 μm [59]. Total deposition efficiency was computed by calculating the number of particles depositing in the nasal cavity divided by the number of particles released from the nostrils. Then, the deposition efficiency was plotted as a function of the particle impaction factor, $\rho d_{p}^{2}Q$ [59,60], where ρ is the density of inhaled particles, *d*_*p*_ is the particle aerodynamic diameter, and *Q* is the volumetric flow rate.

## 3. Results

### 3.1. Morphology

The nasal airway geometries (total volume and surface area) are shown in (Table 1 and Fig. 2). The overall geometric characteristics were reasonably consistent with published experimental measurements.

### 3.2. The airflow pattern and dorsal medial (DM) stream

**Fig. 4.**
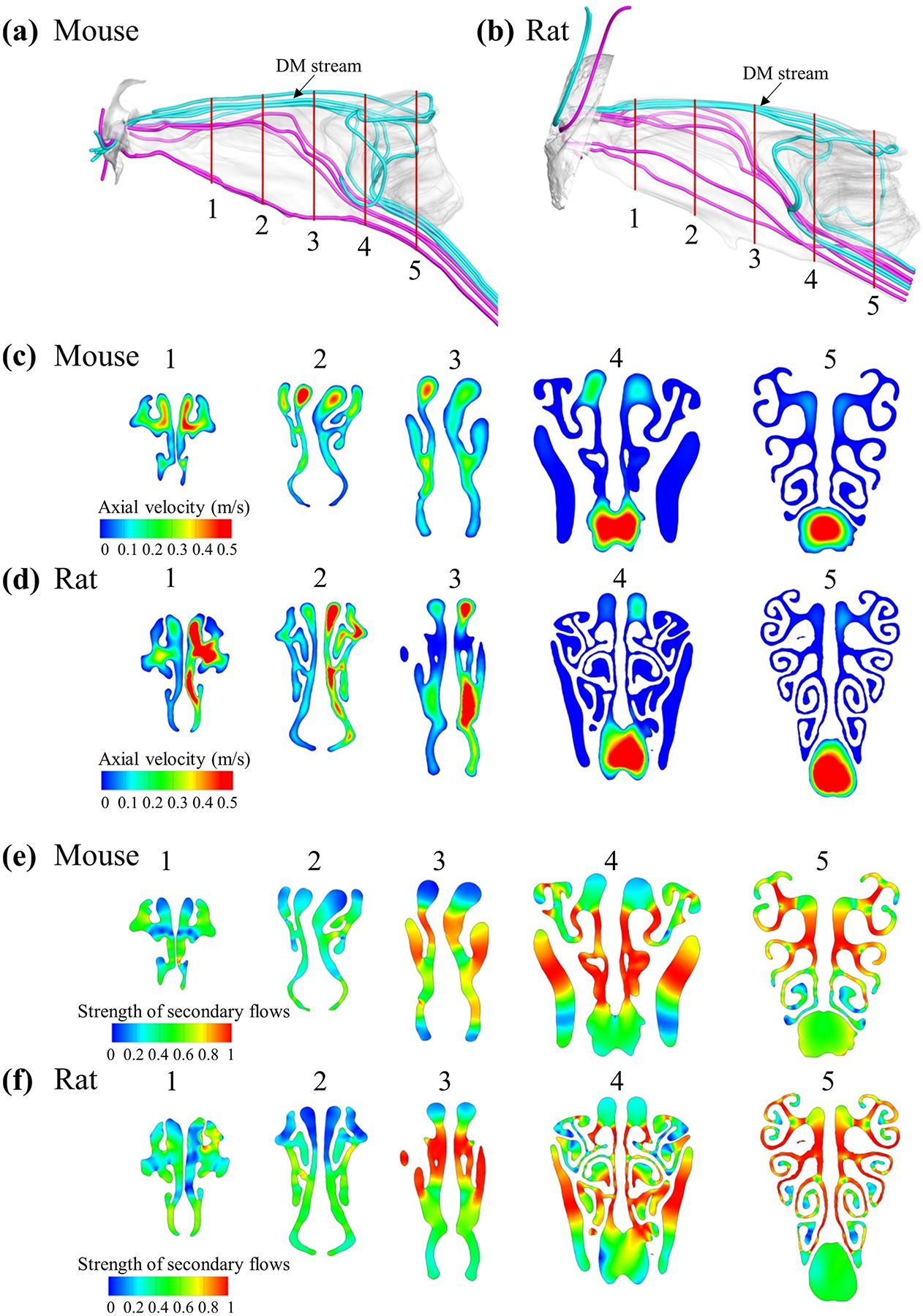
Streamlines for the mouse (a) and the rat (b), where the blue lines represent the DM stream reaching deep into the olfactory region and the purple lines represent the respiratory stream; The axial velocity profile (c,d) and the strength of secondary flows (e,f) on selected coronal planes from the mouse and rat nasal cavities.

As shown in the streamlines and axial velocity (z-direction) contour plots (Fig. 4a–d and supplemental material S1), during inspiration, there is one primary nasal air stream (blue lines in Fig. 4a) that flows dorsal medially entering the ethmoid recess (the olfactory region) followed by diverging S-shaped flow path, before reverse flow direction and exiting the septal window. Aside from the DM stream, the bulk of the respiratory stream (purple lines in Fig. 4a) bypasses the ethmoid recess, flows through the maxillary turbinates, and exists through the ventral pharynx path. Considering the distribution of olfactory epithelium, which is most confined within the ethmoid recess, this may have functional implications related to the olfactory odorant transport. The same phenomenon can be found in previous mammalian nasal models (rat: Fig. 4b, cat [17], and dog [15]).

Five coronal planes were selected to compare the velocity profiles of the mouse and rat models (Fig. 4c–f). Planes #3~#5 were considered to cover the olfactory region. Plane #5 best represents the complexity of the Ethmoid Recess (ER), i.e., the olfactory, of the mouse and rat models. As shown in Fig. 4c–f, the rat model visually has a more complex geometry than the mouse. Especially at plane #5, the ER region, the ethmoid scrolled structure is more extended in the rat model.

Fig. 4e,f shows the contour of the five coronal planes’ secondary flow (*SS*) distributions. Similar *SS* patterns were found in the rat and mouse models: (1) The strength of secondary flows in the ethmoid recess (planes #4 and #5) was much stronger than that in the anterior region (planes #1 and #2). The low axial flow rates of lateral ethmoid recess can be misleading (as shown in Fig. 4c,d), as the strength of secondary flow is very strong in this region (Fig. 4e,f); (2) in the ethmoid recess (see plane #5 in Fig. 4e,f), most robust secondary flows were concentrated in the airways that connect the dorsal medial (DM) region to the lateral coiled channels, serving to divert airflow out of DM stream into lateral and ventral channels during inspiration. The strength of secondary flow is feeble in the DM and nasopharynx regions where axial velocity was high. The average strength of secondary flows in the olfactory region on plane #5 (Fig. 4e,f) in the mouse (0.72) is slightly weaker than that of the rat (0.76), which implies that the rat may have a slightly better lateral olfactory odorant transport rate than the mouse.

Overall, the airflow patterns of axial and secondary flow in the rat and mouse models suggest that the scroll-shaped olfactory turbinates, extending from central to lateral olfactory, serve to redirect airflow and odorants from the dorsal medial stream into lateral channels.

### 3.3. Odorant absorption in the rat and mouse nasal cavities

**Fig. 5.**
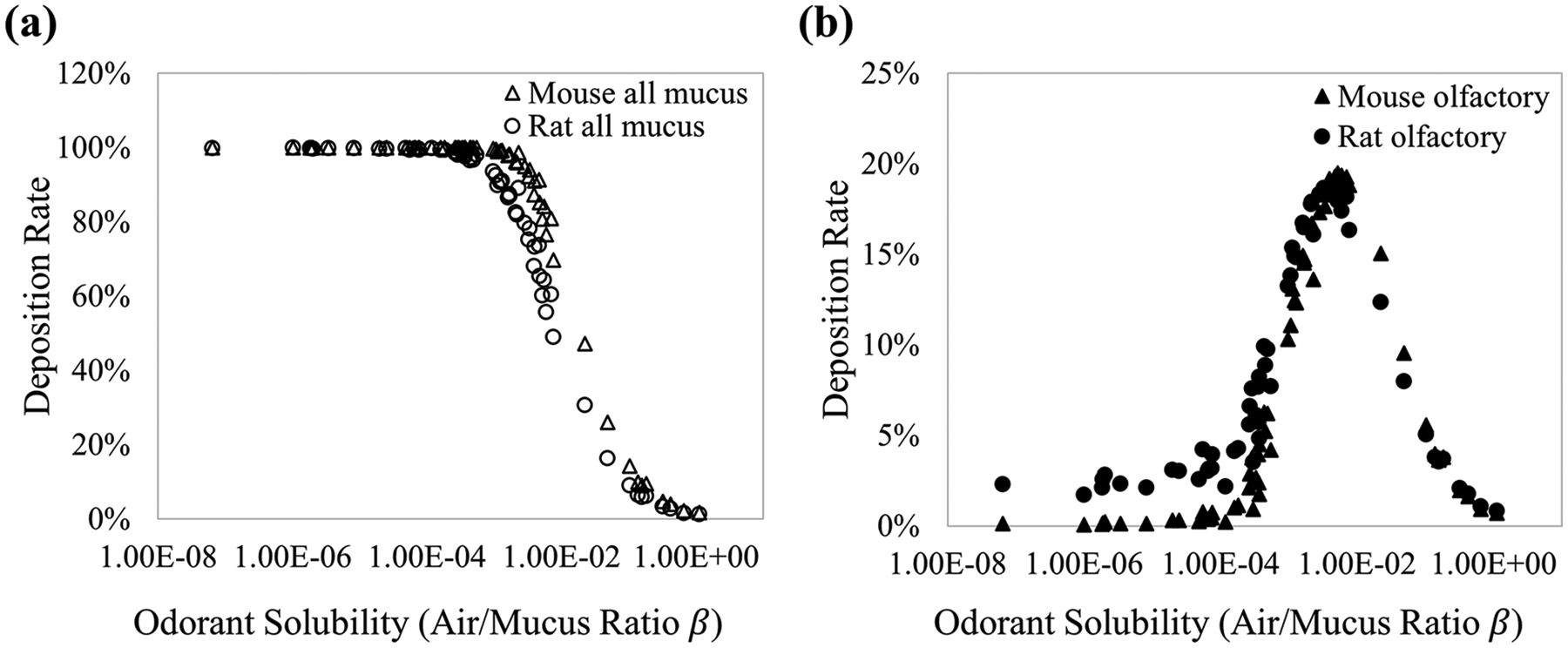
Deposition rates of various odorants on total nasal mucosa (a) and olfactory mucosa (b).

This study simulated the process of odor absorption onto the nasal mucosa. Fig. 5 shows the deposition rates of various odorants on the total nasal mucosa (Fig. 5a) and olfactory mucosa (Fig. 5b). The odor deposition rate is the ratio of the absorbed odor to the total inlet incoming odor concentration. The mouse and rat models have similar deposition curves overall, but the rat model has a slightly higher olfactory deposition rate at high to intermediate odorant solubility range (β =1e-8 to 1e-2) than the mouse model (Fig. 5b), potentially due to the better lateral olfactory odorant transport rate as shown before.

**Fig. 6.**
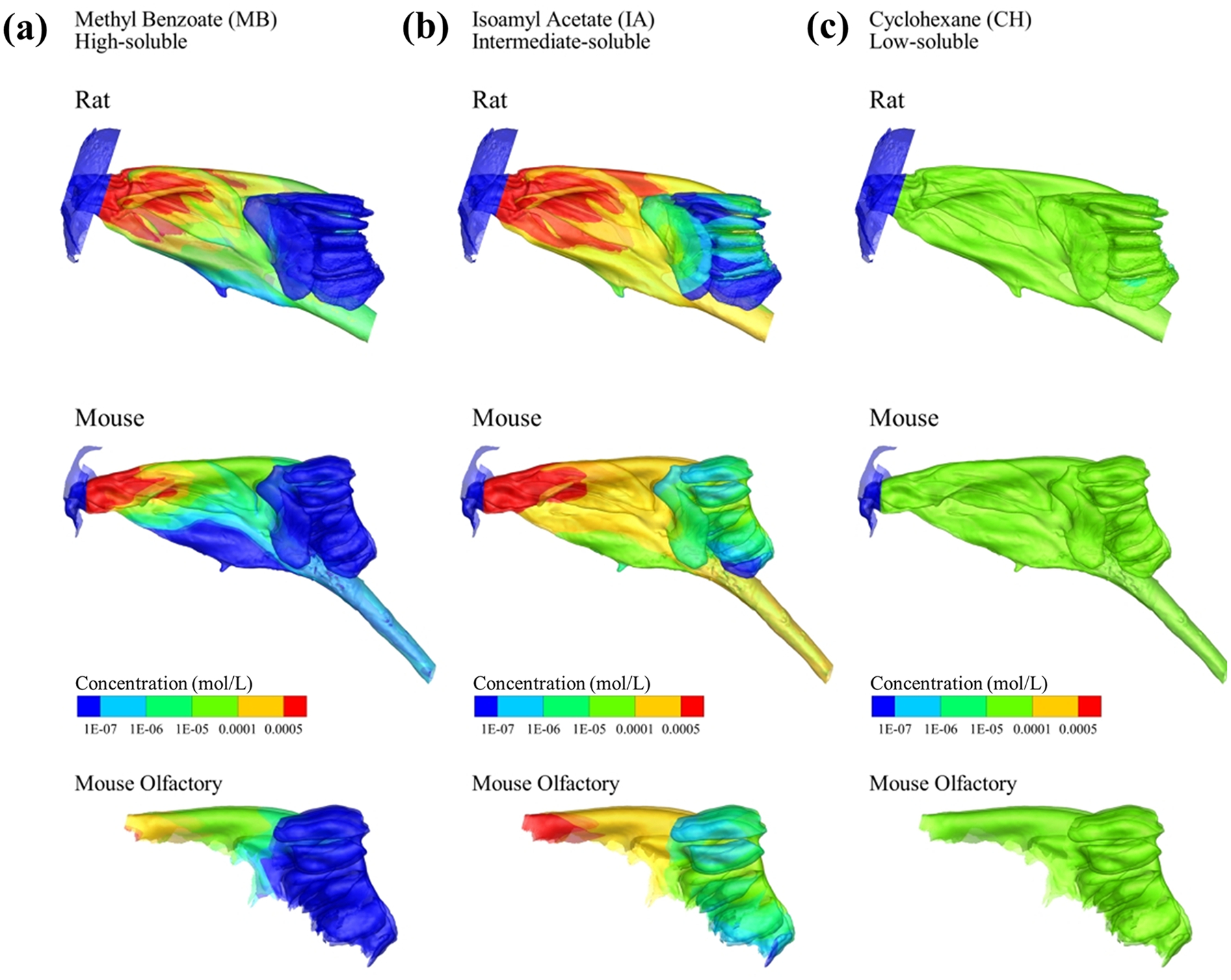
Odor absorption map of three odorants: (a) a very mucosa-soluble Methyl Benzoate (MB), (b) an intermediate-soluble Isoamyl Acetate (IA), and (c) a low-soluble Cyclohexane at the mouse and rat nasal mucosal surfaces and the olfactory region.

Fig. 6 displays the log-scale absorption map of three odorants during a restful breathing condition: a very mucosa-soluble Methyl Benzoate (MB), an intermediate-soluble Isoamyl Acetate (IA), and a low soluble Cyclohexane (CH, see S2 Table for the odorants’ properties). The results are similar to those obtained previously [17,40]; the intermediate-soluble odorant (Fig. 6b) has the highest olfactory absorption in both rat and mouse models. High-soluble (Fig. 6a) odorants were depleted quickly in the anterior nasal cavity. This mechanism also explains the Gaussian-like olfactory deposition curve in Fig. 5b.

The absorption maps for the rat and mouse models are generally similar but with more absorption gradients in the olfactory region of the rat model, especially for the intermediate-soluble odorants, potentially due to the different ethmoid turbinate complexities (as shown in plane #5 in Fig. 4e,f).

### 3.4. Gas chromatography efficiency in the mouse and rat olfactory regions

**Fig. 7.**
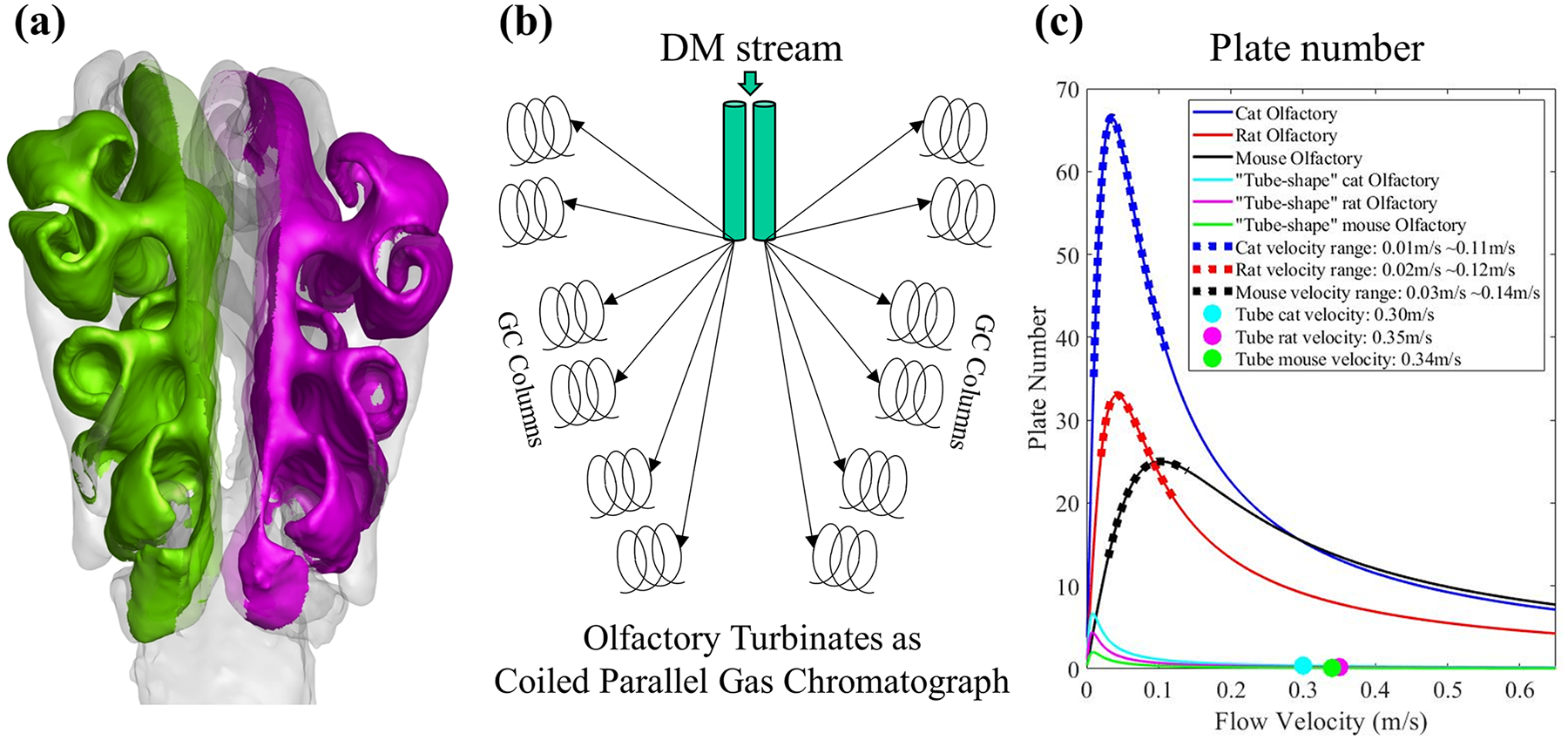
(a) The olfactory turbinates of the mouse. (b) A gas chromatograph system with parallel lateral ethmoid coils, each serving as a CG column, fed by high-speed DM airflow. (c) The theoretical plate number for the cat [17], rat, mouse, and the “straight tubes” olfactory regions. The dots represent the range of airflow velocity in the olfactory areas of different models.

Based on our previous work [17]. The scroll-shaped olfactory turbinates (Fig. 7a), extending from central to lateral, could be analogous to a coiled parallel gas chromatograph (GC) system (Fig. 7b). The DM stream is separated into multiple parallel lateral paths through the olfactory turbinates, each parallel path serving as a parallel GC column. With the theoretical plate number concept, we evaluated the GC efficiency for the mouse and rat olfactory. A higher theoretical plate number represents a greater capacity to differentiate odorants [61,62]. As shown in Fig. 7c, the rat olfactory (red curve) has a higher peak plate number (Npeak=33) than the mouse (black curve, Npeak=25); however, both are significantly lower than previous data of the cat (blue curve). This may be due to the different complexity of ethmoid turbinate, different strengths of lateral flow, as well as length of flow path inside the olfactory system (Eq. (3)). To emphasize the impact of the complexity of the olfactory system on GC efficiency, we then replace the complex olfactory system by a simple amphibian-like “elongated-tube” olfactory system for both the mouse and rat models and calculated corresponding GC plate number (green and magenta curves in Fig. 7c). Consistent with our previous cat model, the peak plate number dropped dramatically to Npeak=2 and 4 for the mouse and rat models, respectively. Furthermore, the velocity in an “elongated-tube” olfactory directly fed from the dorsal medial stream (~0.35 m/s; Fig. 7c, green and purple dots) is much higher than the optimal velocity which could obtain peak plate number in this system. Thus, the actual plate number calculated from the “elongated-tube” olfactory system was further reduced (0.1 for the mouse, 0.2 for the rat), less than ~1/100th of the actual olfactory region. In contrast, the velocity in the rat and mouse olfactory systems are low enough (0.02–0.12 m/s for the rat, 0.03–0.14 m/s for the mouse; Fig. 7c, red and black dots) to achieve the peak plate number, due to the parallel flow paths. Current research reaffirmed that the complex olfactory turbinates might serve as a highly efficient gas chromatograph system, enhancing odor processing capability over a simple “elongated-tube” nose.

Further comparing the plate number curves between the mouse and rat (Fig. 7c), we found that the rat GC efficiency is more sensitive to velocity than the mouse, which indicates that although the rat has a higher peak plate number, the mouse could have a better GC efficiency on a broader velocity range, even overlapping with that of the cat at high-velocity range. Potentially, the configuration in the mouse olfactory pathways may help the odorant deposition and adapt better to the variability of velocity under a wide range of sniffing conditions.

### 3.5. Particle deposition

**Fig. 8.**
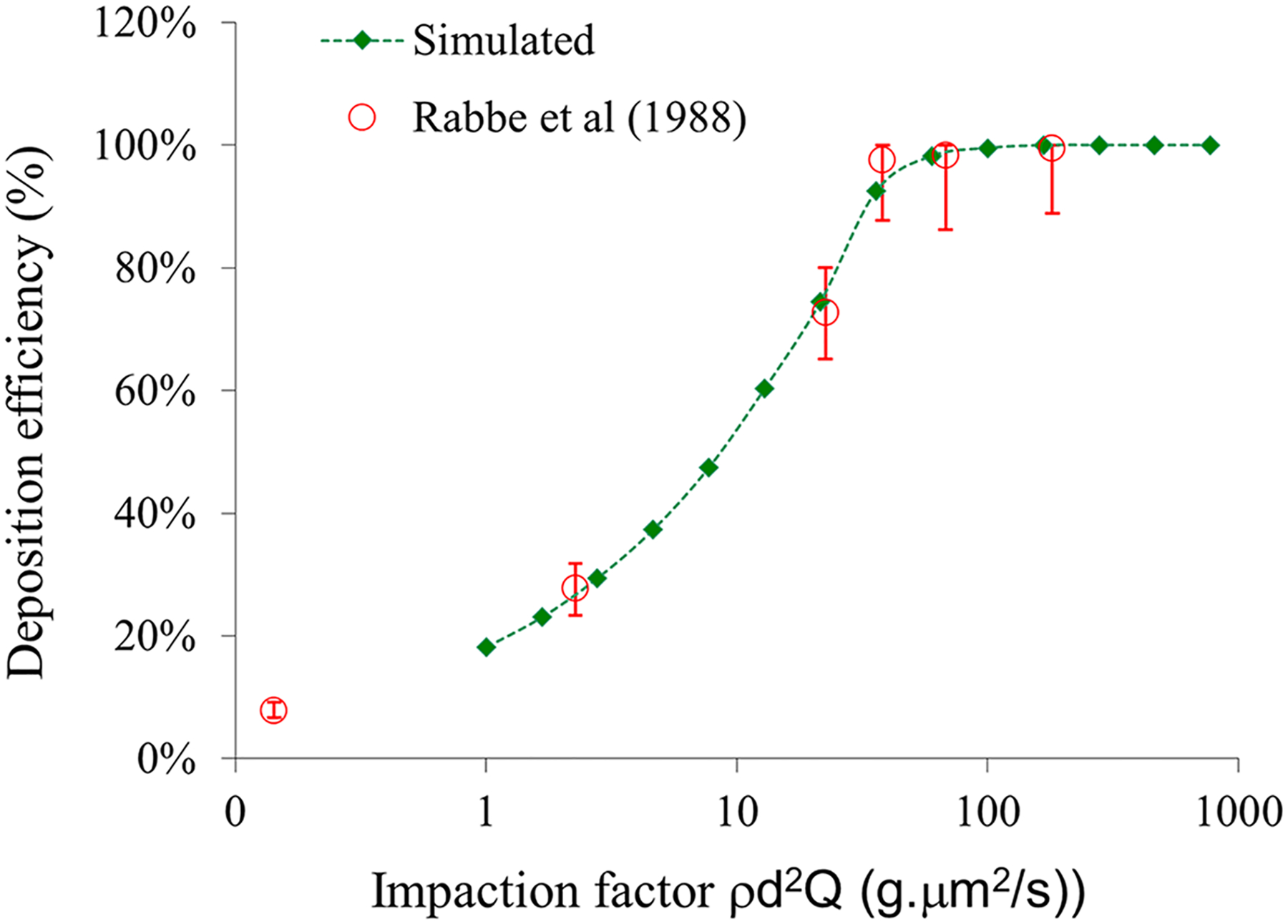
The comparison of the CFD model predicted nasal deposition efficiency with experimental data as a function of the impaction factor.

Particle deposition simulations were conducted using the Discrete Phase Model in FLUENT (ANSYS, Inc., Canonsburg, PA) under a steady restful breathing flow rate (25 mL/min). The deposition efficiency was plotted as a function of the impaction factor, which is a derivation of the Stokes number, as shown in Fig. 8 (green square) [60]. To validate our numerical simulation, we compared the deposition efficiency curve with published experimental data [28] (red circles in Fig. 8).

Particle transport and deposition depend on the airflow that carries the particles and the particle size. As airflow rate or particle size increases, the inertia dominates the movement, and the deposition efficiency increases. As shown in Fig. 8, when the impaction factors are greater than 100, or the particle aerodynamic diameters are greater than 15 μm under the current flow rate, the deposition efficiency reaches over 99 %. This implies that the particles with aerodynamic diameters greater than 15 μm will primarily deposit within the mouse nasal cavity. Compared to the previous CFD simulation in the human nasal cavity, which found that 18 μm particles under restful breathing were predicted to deposit with greater than 99 % efficiency [60], the mouse model has a slightly different particle size range than the human for similar deposition efficiency.

## 4. Discussion

The mouse is one of the most critical animal models used extensively in biomedical research [21–29]. However, limited nasal airflow analysis for mice exists [24,35–39], potentially due to its small size and complex peripheral olfactory structure. The advance in contrast-enhanced high-resolution micro-CT imaging (isotropic resolution 10.5 μm/pixel) has enabled us to visualize the 3D exquisitely intricate architectures of the mouse nasal airways in great detail. The image-based computational fluid dynamic modeling and simulation of respiration and olfactory airflow path further quantitatively revealed the characteristics of the mouse nasal airflow patterns that haven’t been detailed before and deepened our understanding of its role in supporting the physiological functions of the nose. The structural and aerodynamic similarities and uniqueness between mice and rats provided valuable insights into their sense of olfaction and their multifaceted adaptations to environments.

During inspiration, two major airflow regimes were formed in the mouse and rat models (Fig. 4a,b) - one flows dorsal medially (DM), entering the olfactory region. At the same time, the other bypasses the ethmoid recess and flows over the respiratory turbinates ventrally to the pharynx. The existence of DM stream has been widely reported in many mammalian species [14,17,18]. It can potentially allow quick delivery of odors to the olfactory region. The airflow in the lateral coiled ethmoid turbinate region likely recirculates from the DM stream. We verified this by using a novel analysis of secondary flow distributions as shown in Fig. 4e,f, where the strength of secondary flows (*SS*) is most robust in the ethmoid recess (olfactory region, plane #3~#5, Fig. 4e,f) and radiates from the airways that connect the dorsal medial (DM) region to the lateral coiled channels, which serve to divert airflow out of DM airway into lateral olfactory channels during inspiration.

Interestingly, the rat nasal airflow has slightly stronger secondary flows than the mouse nasal airflow (plane #5, Fig. 4e,f). This might lead to a slightly better olfactory odorant transport efficiency in the rat olfactory system than in the mouse olfactory system since more airflow (with odorant molecules) could be transported from the dorsal medial region to the lateral coiled channels. The deposition rate curves of the mouse and rat olfactory system (Fig. 5b) support this finding, where the rat has a slightly higher deposition rate than the mouse for the high to intermediate-soluble odorants. From the odor absorption maps (Fig. 5b), we noticed some differences between the rat and mouse nasal models. The absorption gradient is more robust in the anterior region of the mouse model, whereas the absorption gradient is more evident in the olfactory region of the rat model. This could be because the rat model has a more complex ethmoid turbinate structure (see planes #4 and #5, Fig. 4c,d), which might lead to more gradient in odor deposition in the olfactory region.

All of the outcomes from the traditional computational fluid dynamic analysis so far implicated that the rat model might have slightly better olfactory odorant transport efficiency than the mouse model. To further explore a quantitative comparison of the odorant transport efficiency between the mouse and rat olfactory models, we applied a novel gas chromatograph (GC) efficiency analysis based on theoretical GC plate numbers [55,56,62] that was previously developed for a cat nasal model [17]. Not surprisingly, the rat model has a slightly higher peak plate number than the mouse (Npeak= 33 and 25, respectively); however, both are significantly lower than the cat (Npeak=67). The different GC plate numbers suggested that the levels of complexity in the ethmoid turbinate anatomy and aerodynamics may have crucial functional relevance in what we termed the “parallel coiled chromatograph” hypothesis. The ethmoid turbinate forms parallel coils that offer longer combined airflow pathways within the confined olfactory region while feeding collectively from the high-speed dorsal medial stream so that total odor sampling speed is not sacrificed. Despite the mouse (or rat) ethmoid turbinates being much simpler than that of the cat, they still offer much better efficiency than an amphibian-like “elongated” olfactory system that fits within the same physical boundary and lacks the parallel coiled feature [63]. Consistent with our previous results, the amphibian-like “elongated” olfactory systems’ peak plate numbers dropped dramatically to Npeak=2 and 4 for the mouse and rat, respectively. The parallel feature in the mouse and rat olfactory turbinates also reduces airflow speed within each coil, which is critical to achieving the optimal plate number. In contrast, the airflow velocity in the “elongated olfactory tube” (~0.35 m/s) is far too high to achieve the optimal plate number. Thus, we further demonstrated that complex parallel coiled olfactory turbinate is likely a common feature among mammalian species to increase olfactory odorant processing efficiency (while limited by the physical skull size). During the comparison, we noticed that the gas chromatograph efficiency in the mouse model, while being lower than that of the rat, is also less sensitive to the airflow velocity than that of the rat, especially near the peak plate number region, which implicates the mouse could have high gas chromatograph efficiency over a broader airflow velocity range than the rat. The reason for this phenomenon could be the narrower cross-section area in the mouse olfactory pathways (Fig. 1), which may help the odorant deposition and reduce the impact of velocity variation on gas chromatograph efficiency or the odorant transport efficiency.

Limitations of this study include, besides the experimental particle deposition data that validated the computational data exceptionally well (Fig. 8), there is a lack of physiological data in odor transport and deposition to compare with computational analysis.

## 5. Conclusion

Although the overall aerodynamics behaves similarly in rat and mouse nasal models, there are some moderate differences in odorant transport due to the structural differences in their olfactory turbinate structure. Under restful breathing conditions, the rat model may have slightly better odorant transport efficiency than the mouse model due to its more complex ethmoid (olfactory) recess structure. However, the mouse nasal structure could reduce the impact of velocity variation on the gas chromatograph efficiency and potentially perform better in a broader range of airflow conditions (for example, the strong sniffing rate). The different odorant transport in the noses of rats and mice could potentially affect their food selection and other behavioral traits, which require additional investigation. Future research may further shed light on the intricate relationship between nasal structure and function and the complex adaptations of mammalian species to various ecological environments.

## Supplementary Material

Supplement word document

supplement spreed sheet

## Figures and Tables

**Table 1:** The nasal geometric characteristics of our CFD model and experimental measurements.

	Surface area (cm^2^)	Volume (mL)	References
B6 mouse	2.90	0.0291	Present study
B6C3F1 mouse (16 weeks, 33 g)	2.89±0.13	0.0315±0.0021	Gross et al. (1982)
B6C3F1 mouse (7 weeks, 30 g)	2.77±0.16	0.0325±0.0032	Gross et al. (1982)
