# Supplementary material for: Is the mouse nose a miniature version of a rat nose? A computational comparative study
Source: Comput Methods Programs Biomed. Author manuscript; Available in PMC 2025 May 31. (PMC12125470; doi:10.1016/j.cmpb.2024.108282)
Supplement: supplement spreed sheet [file NIHMS2084474-supplement-supplement_spreed_sheet.docx]

***1. Airflow pattern analysis***

Equations for Womersley number $W_{0}$ [1] and the Strouhal number $S$ [2]:

$W_{0}=R\sqrt{\left( \frac{2\pi f}{\nu} \right)}, S=\frac{2\pi fL}{U}$ (1)

Reynolds number:

$Re=\frac{UR}{\nu}$ (2)

where *R* is the hydraulic diameter (the external naris’s diameter), $f$ is the sniffing frequency, $\nu$ is the air kinematic viscosity, *L* is the characteristic length of the nasal cavity (axial length from the external nares to the nasopharynx), and *U* is the average velocity [3]. The Womersley number is a dimensionless ratio of the unsteady inertial forces in relation to viscous forces. Meanwhile, the Strouhal number is derived from the ratio of the steady boundary layer thickness to the Stokes layer thickness [4], representing the ratio of unsteady inertial forces or local acceleration to the convective inertial forces.

***2. Theoretical plate concept***

The theoretical plate concept was adopted to describe the efficiency of the gas chromatography columns [3]. We used the Golay equation [5,6] to evaluate the plate height (*H*) of the GC systems:

$H=\frac{2D_{a}}{\bar{u}}+\frac{2k^{'}d^{2}\bar{u}}{3{(1+k^{'})}^{2}D_{m}}+\frac{(11k^{'2}+6k^{'}+1)d_{c}^{2}\bar{u}}{96{(1+k^{'})}^{2}D_{a}}$ (3)

where $\bar{u}$ is the averaged linear velocity, $d_{c}$ is the averaged channel width of the column, and $k^{'}$ is the capacity factor, or retention factor, which can be calculated by [7]:

$k^{'}=\frac{C_{m}}{C_{a}}*\frac{V_{m}}{V_{a}}$ (4)

here, $V_{m}$ and $V_{a}$ are the stationary and mobile phase volumes. The mobile phase is the airway in the paths, and the stationary phase is the mucus layer, assumed to be 30 µm thick [8], coated on the inner wall of each column. $C_{m}$ and $C_{a}$ are the molar concentrations of the odor in the stationary phase and mobile phase, respectively. The inlet and outlet of the olfactory section were defined on planes #3 and #5, shown in Fig 4a, to acquire the averaged inlet and outlet concentration, $C_{in}$ and $C_{out}$:

$C_{m}=C_{in}-C_{out}, C_{a}=C_{out}$ (5)

***3. The velocity profile on Sagittal planes of the mouse and rat models***

***
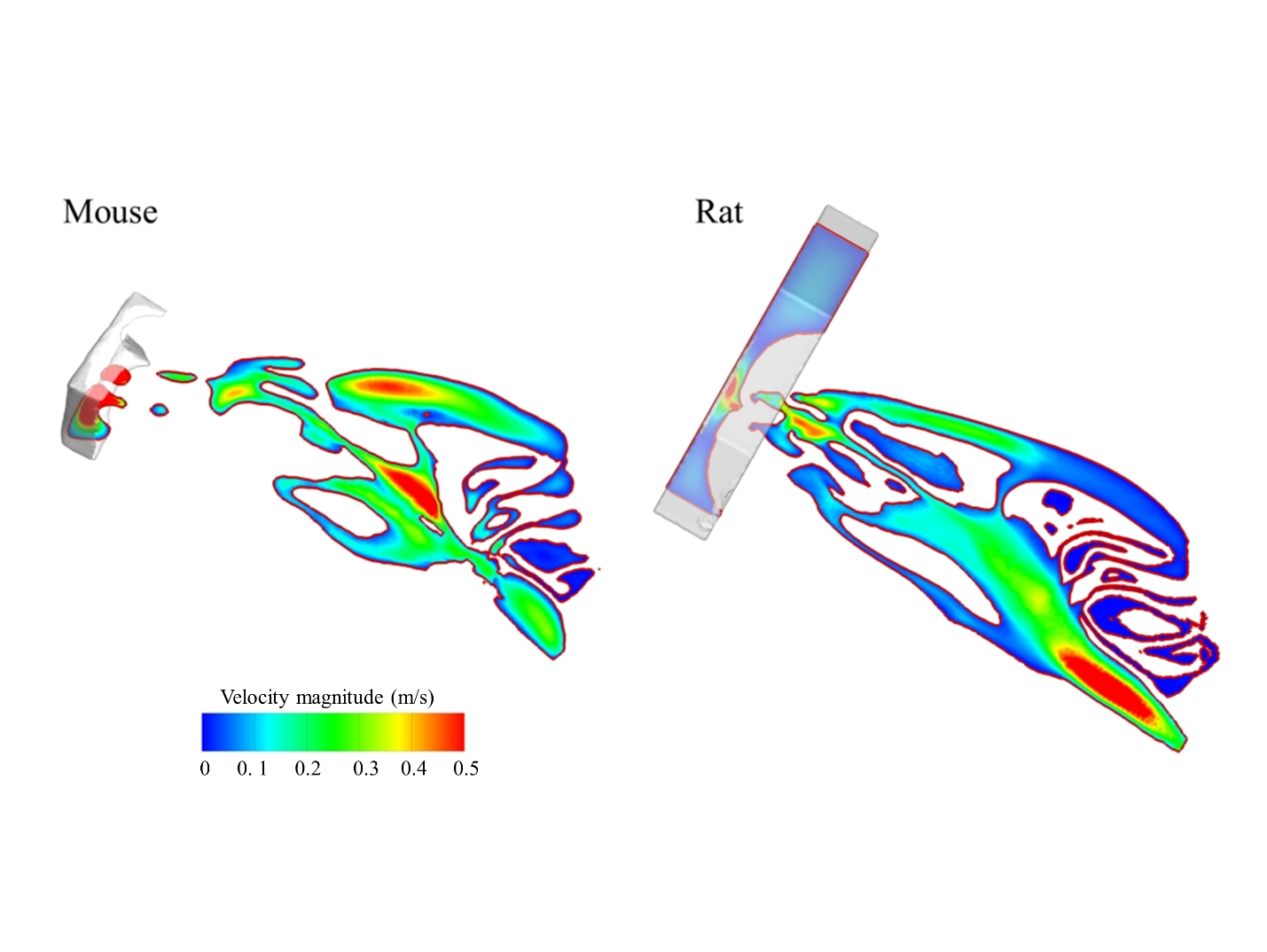
***

***References:***

1. Womersley JR. Method for the calculation of velocity, rate of flow and viscous drag in arteries when the pressure gradient is known. The Journal of Physiology. 1955;127: 553–563. doi:10.1113/jphysiol.1955.sp005276

2. Schroter RC, Sudlow MF. Flow patterns in models of the human bronchial airways. Respiration Physiology. 1969;7: 341–355. doi:10.1016/0034-5687(69)90018-8

3. Wu Z, Jiang J, Lischka FW, McGrane SJ, Porat-Mesenco Y, Zhao K. Domestic cat nose functions as a highly efficient coiled parallel gas chromatograph. Graham LJ, editor. PLoS Comput Biol. 2023;19: e1011119. doi:10.1371/journal.pcbi.1011119

4. Pedley TJ. Pulmonary Fluid Dynamics. Annu Rev Fluid Mech. 1977;9: 229–274. doi:10.1146/annurev.fl.09.010177.001305

5. Golay MJE. Height equivalent to a thoretical plate of an open tubular column lined with a porous layer. Anal Chem. 1968;40: 382–384. doi:10.1021/ac60258a034

6. Ishii D, Takeuchi T. Open Tubular Capillary LC. Journal of Chromatographic Science. 1980;18: 462–472. doi:10.1093/chromsci/18.9.462

7. Ettre LS. Nomenclature for chromatography (IUPAC Recommendations 1993). Pure and Applied Chemistry. 1993;65: 819–872. doi:10.1351/pac199365040819

8. Getchell T, Margolis F, Getchell M. Perireceptor and receptor events in vertebrate olfaction. Progress in Neurobiology. 1984;23: 317–345. doi:10.1016/0301-0082(84)90008-X
